# Noise-induced switches in network systems of the genetic toggle switch

**DOI:** 10.1186/1752-0509-1-50

**Published:** 2007-11-15

**Authors:** Junwei Wang, Jiajun Zhang, Zhanjiang Yuan, Tianshou Zhou

**Affiliations:** 1 State Key Laboratory of Biocontrol and Guangzhou Center for Bioinformatics, School of Life Science, Sun Yat-Sen University, Guangzhou 510275, P.R. China; 2 School of Mathematics and Computational Science, Sun Yat-Sen University, Guangzhou 510275, P.R. China

## Abstract

**Background:**

Bistability, the capacity to achieve two distinct stable steady states in response to a set of external stimuli, arises within biological systems ranging from the *λ *phage switch in bacteria to cellular signal transduction pathways in mammalian cells. On the other hand, more and more experimental evidence in the form of bimodal population distribution has indicated that noise plays a very important role in the switching of bistable systems. However, the physiological mechanism underling noise-induced switching behaviors remains to be fully understood.

**Results:**

In this paper, we investigate the effect of noises on switching in single and coupled genetic toggle switch systems in *Escherichia coli*. In the case of the single toggle switch, we show that the multiplicative noises resulting from stochastic fluctuations in degradation rates can induce switching. In the case of the toggle switches interfaced by a quorum-sensing signaling pathway, we find that stochastic fluctuations in degradation rates inside cells, i.e., intracellular noises, can induce synchronized switching, whereas the extracellular noise additive to the common medium can not only entrain all the individual systems to switch in a synchronous manner but also enhance this ordering behavior efficiently, leading a robust collective rhythm in this interacting system.

**Conclusion:**

These insights on the effect of noises would be beneficial to understanding the basic mechanism of how living systems optimally facilitate to function under various fluctuated environments.

## Background

Both natural and synthetic gene networks regulated at the level of gene transcription and translation are capable of exhibiting complex dynamic behaviors [1-3]. Among the various patterns of regulation associated with nonlinear kinetics of gene regulatory networks, bistability allows a graded signal to be turned into a discontinuous evolution of the system along several possible distinct signaling pathways which can be either reversible or irreversible [4-7]. It has been shown that even relatively simple signaling networks have the potential to produce bistability [2-6]. A system is termed bistable if it can switch between two distinct stable steady states but cannot rest in intermediate states under the excitation of external stimuli. Biological examples of bistable systems include the *λ *phage lysis-lysogeny switch [8,9], several mitogen-activated protein kinase cascades in animal cells [10-12], and cell cycle regulatory CI circuits in *Xenopus *and *Saccharomyces cerevisiae *[13,14]. Usually, bistable systems in the biological context are thought of as those involved in the generation of switch-like biochemical responses [10,11,15], the establishment of cell cycle oscillations and mutually exclusive cell cycle phases [14], the production of self-sustaining biochemical "memories" of transient stimuli [16,17], or the rapid lateral propagation of receptor tyrosine kinase activation [18]. In spite of their simple dynamic behaviors, bistable systems are building blocks of larger regulatory elements: genetic networks and signaling cascades. Moreover, the pathways by which they operate would be passed on from generation to generation. Understanding their stability and characteristics is therefore fundamental yet important.

Recently, increasingly experimental evidence in the form of bimodal population distribution indicates that noise plays a very important role in the switching of bistable systems, e.g., the genetic toggle switch and lactose operon systems [6,19,20]. First, noise exists extensively in biological systems with a small number of molecules (as is the case in transcriptional regulation systems) due to the intrinsically stochastic nature of biochemical reactions involved or because of environmental fluctuations. It has been proposed that noise in the form of random fluctuations arises in biological networks in one of two ways, namely internal noise or external noise [21,22]. Second, since living systems are usually optimized to function in the presence of stochastic fluctuations [23], the biochemical networks must withstand considerable variations and random perturbations of biochemical parameters [24]. Such a property of biological systems is known as robustness. On the other hand, some biological organisms can exploit environmental fluctuations and/or various intracellular noises to communicate with other cells or introduce diversity into a population of cells. For example, extracellular noise can act as a compensating signal source to enhance an integrated exchange of information and force all the cells to be stochastically synchronized, thus attaining intercellular communication in a synchronous manner [25]. Another example is shown when an infected *λ *phage determines a pathway [8]. Such dual ("negative" and "positive") effects of noises might be actually exploited by realistic living organisms or cells to positively facilitate some functions.

The constructive role of noise in mediating signaling transduction of cells in response to environmental changes or external signals has also been reported. For example, in Metazoan, individual steady states (attractors) existing in bistable or multistable systems correspond to particular functional cell states or cell types [26,27]. Signal transduction machineries can efficiently convey external changes to the gene regulation apparatus in order to switch between coherent genetic programs. More interestingly, Kashiwagi *et al*. [28] showed that in the absence of signal transduction, switching to the appropriate attractor state expressing the genes that afford adaptation to the external condition can still occur. They found that in a synthetic bistable gene switch in *E. coli *in which mutually inhibitory operons govern the expression of two genes required in two alternative nutritional environments, cells reliably select the "adaptive attractor" driven by noises from gene expression. However, the physiological mechanism hidden in these phenomena, in particular the one for noise-induced switch-like behaviors has not been well explored yet.

There have been some studies which have explored the role of noises in the transitions of the genetic toggle switch [6,19,21,29-33]. For example, Tian and Burrage [29], based on the Poisson *τ*-leap method which is an improved version of Gillespie' algorithm [34], developed a general methodology for introducing noise into deterministic models described by ordinary differential equations (ODE) in a very simple manner and demonstrated the power of this technique by analyzing the stochastic behaviors of the genetic toggle switch interfaced with either the SOS signal pathway or the quorum-sensing signal pathway. They showed that the noise introduced in such a manner can induce switching between two alternative states in the genetic toggle switches, successfully realizing experimental results showing bimodal population distributions [6,19]. We note that such a noise is actually internal noise since the theoretical background of introducing such stochastic models comes from the *τ*-leap methods that link the stochastic simulation of biochemical reaction system to the Euler method for solving ODEs via the mean. However, whether or not other kinds of noises, such as additive or multiplicative noises to be introduced in the paper, is enough to switch in the genetic toggle switch or its population from one steady state to the other has not been investigated yet. This is precisely what we will address here. Since there are a few ways to introduce external noise, e.g., if the number of molecules is sufficiently large, one may adopt the Langevin approach to give a stochastic differential equation, here we more directly introduce noises by considering stochastic fluctuations in degradation rates in the genetic toggle switch or environmental fluctuations in a population of the toggle switches interfaced by a quorum-sensing signaling pathway. And we show that multiplicative noises introduced as in [21,22] in the single toggle switch system can induce switching between alternative stable steady states, and that the extracellular noises in the coupled toggle switch systems can not only force every toggle switch to switch between the two stable steady states, but also attain and even enhance a synchronized switching. In the coupling case, we also show that except for the ability to induce synchronized switching, intracellular noises can play a role of amplifier for gene expression in every toggle switch. In particular, when the noises are interacted with an injected sinusoidal signal (or stimulus) in the environment, their effects become more notable, e.g., a more robust collective rhythm can be achieved.

We point out that although the result that noise can induce coherent resonance is not novel from a view of physics [35,36], the effect of external noises in biologically plausible systems, in particular in multicellular bistable systems with signaling pathways, is rarely investigated [29], and most of previous biological results about this aspect are in experiments works [19]. Our investigation is a significant try forward understanding the basic mechanism of external noise-induced switching in realistic yet complex organisms.

## Methods and Results

### Case 1: The single genetic toggle switch

#### Model

As is well known, the toggle switch is composed of two transcription factor proteins [6]: **LacI **and *λ ***CI **that are encoded by genes *lacI *and cI, respectively. The synthesis of the two repressor proteins is regulated in such a way that the expression of the *cI *and *lacI *genes is mutually exclusive: The promoter that controls the expression of *cI *is attenuated by the protein **LacI **while the promoter that controls the expression of *lacI *is attenuated by the protein *λ ***CI**. Thus, a cell can be either in a state where *λ ***CI **is abundant and **LacI **scare (the *λ ***CI **on the state) or in a state where **LacI **is abundant and *λ ***CI **scare (the **LacI **on the state). The protein dynamics is described by the following equations [6]:

dxdt=α11+yn1−d1x+γ1
 MathType@MTEF@5@5@+=feaafiart1ev1aaatCvAUfKttLearuWrP9MDH5MBPbIqV92AaeXatLxBI9gBaebbnrfifHhDYfgasaacPC6xNi=xI8qiVKYPFjYdHaVhbbf9v8qqaqFr0xc9vqFj0dXdbba91qpepeI8k8fiI+fsY=rqGqVepae9pg0db9vqaiVgFr0xfr=xfr=xc9adbaqaaeGacaGaaiaabeqaaeqabiWaaaGcbaqcfa4aaSaaaeaacqWGKbazcqWG4baEaeaacqWGKbazcqWG0baDaaGccqGH9aqpjuaGdaWcaaqaaGGaciab=f7aHnaaBaaabaGaeGymaedabeaaaeaacqaIXaqmcqGHRaWkcqWG5bqEdaahaaqabeaacqWGUbGBdaWgaaqaaiabigdaXaqabaaaaaaakiabgkHiTiabdsgaKnaaBaaaleaacqaIXaqmaeqaaOGaemiEaGNaey4kaSIae83SdC2aaSbaaSqaaiabigdaXaqabaaaaa@451C@

dydt=α21+xn2−d2y+γ2
 MathType@MTEF@5@5@+=feaafiart1ev1aaatCvAUfKttLearuWrP9MDH5MBPbIqV92AaeXatLxBI9gBaebbnrfifHhDYfgasaacPC6xNi=xI8qiVKYPFjYdHaVhbbf9v8qqaqFr0xc9vqFj0dXdbba91qpepeI8k8fiI+fsY=rqGqVepae9pg0db9vqaiVgFr0xfr=xfr=xc9adbaqaaeGacaGaaiaabeqaaeqabiWaaaGcbaqcfa4aaSaaaeaacqWGKbazcqWG5bqEaeaacqWGKbazcqWG0baDaaGccqGH9aqpjuaGdaWcaaqaaGGaciab=f7aHnaaBaaabaGaeGOmaidabeaaaeaacqaIXaqmcqGHRaWkcqWG4baEdaahaaqabeaacqWGUbGBdaWgaaqaaiabikdaYaqabaaaaaaakiabgkHiTiabdsgaKnaaBaaaleaacqaIYaGmaeqaaOGaemyEaKNaey4kaSIae83SdC2aaSbaaSqaaiabikdaYaqabaaaaa@4526@

where *x *and *y *are the concentrations of **LacI **and *λ ***CI**, respectively. *α*_1 _and *α*_2 _are the dimensionless transcription rates in the absence of repressor, *n*_1 _and *n*_2 _are the Hill coefficients, and *d*_*i *_and *γ*_*i *_(*i *= 1,2) are the degradation rates and the basal synthesis rates, respectively. Throughout this paper except for a place, we fix parameters as follows: *α*_1 _= 2.5, *α*_2 _= 5, *d*_1 _= *d*_2 _= 1, *γ*_1 _= *γ*_2 _= 0.5, and the Hill coefficients *n*_1 _= *n*_2 _= 4, which are mainly from [3,19] with slight modifications to maintain bistablity of the genetic toggle switch under considerations.

Cells usually facilitate their functions under various fluctuated environments. It has been shown that stochastic terms in the rate equations can capture the fluctuations in gene expression [21]. These fluctuations result in so-called external noises. In a previous work, the effect of interplay between an external noise and the transcriptional enhancement process has been examined in a single-gene regulatory network of the *λ *phage [21,22]. In contrast to that work which shows that a multiplicative noise resulting from fluctuation of the transcription rate can be used to amplify protein production significantly, here we investigate how an external noise source introduced into degradation rates affects switching in the genetic toggle switch system. In order to introduce such a kind of noise into the model (1) and (2), we adapt an approach in the spirit of Ref. [21]. As the cleavage of repressor is dominant in the process of induction, stochasticity in degradation is more important than other noises in the system [37], and in this case stochasticity in degradation rates of repressors are appropriate to represent external noises. We vary the degradation rates by allowing the parameters *d*_1_and *d*_2 _in the model (1) and (2) to vary stochastically, i.e., *d*_1 _→ *d*_1 _+ *ξ*_1_(*t*) and *d*_2 _→ *d*_2 _+ *ξ*_2_(*t*). In such a manner, we obtain the following stochastic model:

dxdt=α11+yn1−(d1+ξ1(t))x+γ1
 MathType@MTEF@5@5@+=feaafiart1ev1aaatCvAUfKttLearuWrP9MDH5MBPbIqV92AaeXatLxBI9gBaebbnrfifHhDYfgasaacPC6xNi=xI8qiVKYPFjYdHaVhbbf9v8qqaqFr0xc9vqFj0dXdbba91qpepeI8k8fiI+fsY=rqGqVepae9pg0db9vqaiVgFr0xfr=xfr=xc9adbaqaaeGacaGaaiaabeqaaeqabiWaaaGcbaqcfa4aaSaaaeaacqWGKbazcqWG4baEaeaacqWGKbazcqWG0baDaaGccqGH9aqpjuaGdaWcaaqaaGGaciab=f7aHnaaBaaabaGaeGymaedabeaaaeaacqaIXaqmcqGHRaWkcqWG5bqEdaahaaqabeaacqWGUbGBdaWgaaqaaiabigdaXaqabaaaaaaakiabgkHiTiabcIcaOiabdsgaKnaaBaaaleaacqaIXaqmaeqaaOGaey4kaSIae8NVdG3aaSbaaSqaaiabigdaXaqabaGccqGGOaakcqWG0baDcqGGPaqkcqGGPaqkcqWG4baEcqGHRaWkcqWFZoWzdaWgaaWcbaGaeGymaedabeaaaaa@4DB7@

dydt=α21+xn2−(d2+ξ2(t))y+γ2
 MathType@MTEF@5@5@+=feaafiart1ev1aaatCvAUfKttLearuWrP9MDH5MBPbIqV92AaeXatLxBI9gBaebbnrfifHhDYfgasaacPC6xNi=xI8qiVKYPFjYdHaVhbbf9v8qqaqFr0xc9vqFj0dXdbba91qpepeI8k8fiI+fsY=rqGqVepae9pg0db9vqaiVgFr0xfr=xfr=xc9adbaqaaeGacaGaaiaabeqaaeqabiWaaaGcbaqcfa4aaSaaaeaacqWGKbazcqWG5bqEaeaacqWGKbazcqWG0baDaaGccqGH9aqpjuaGdaWcaaqaaGGaciab=f7aHnaaBaaabaGaeGOmaidabeaaaeaacqaIXaqmcqGHRaWkcqWG4baEdaahaaqabeaacqWGUbGBdaWgaaqaaiabikdaYaqabaaaaaaakiabgkHiTiabcIcaOiabdsgaKnaaBaaaleaacqaIYaGmaeqaaOGaey4kaSIae8NVdG3aaSbaaSqaaiabikdaYaqabaGccqGGOaakcqWG0baDcqGGPaqkcqGGPaqkcqWG5bqEcqGHRaWkcqWFZoWzdaWgaaWcbaGaeGOmaidabeaaaaa@4DC3@

where *ξ*_*i*_(*t*) is a random term with zero mean <*ξ*_*i*_(*t*)> = 0, *i *= 1,2. In order to encapsulate rapid random fluctuations, we make the standard requirement that the autocorrelation be "*δ*-correlated," i.e., the statistics of *ξ*_*i*_(*t*) are such that <*ξ*_*i*_(*t*)*ξ*_*j*_(*t'*)> = *Dδ*_*i*, *j*_(*t*-*t*') with *D *proportional to the strength of the perturbation. Thus, the noises are multiplicative in a degradation rate manner, as opposed to those introduced into the transcription rates in the previous work [21].

Except for the mentioned-above reasons of introducing models (3) and (4), there are other considerations. It has been showed that stochastic simulations [30-33,38] play an important role in revealing the design principles underlying the stability of genetic switches. However, the corresponding systems are often very difficult to simulate in a brute-force manner. This is because they can be extremely stable, showing few or no flips during the simulation, e.g., the synthetic genetic toggle switch is a robust bistable system, and internal noise-induced transitions are rare [19,29]. To predict the rate and mechanism of the flipping of these switches, a technique called "forward flux sampling" (FFS) have been proposed, which allows efficient simulation of rare but important events in biochemical networks and has been successfully applied to a genetic toggle switch [31]. In spite of this, most of the relevant studies have considered only the case of internal noises so far, and there are few results about the effect of external noises on switching in the genetic toggle switch. This motivates us to study model (3) and (4). Another motivation is to make comparison between external noise-induced behaviors in the single toggle switch system and in an ensemble of toggle switch systems aforementioned.

#### Results

**Figure 1:**
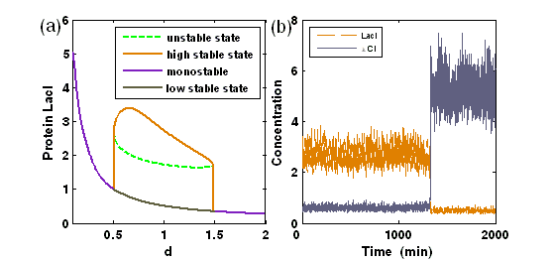
Bifurcation diagram and the effect of external noise on switching in the toggle switch system. (a) Bifurcation plot for the **LacI **concentration vs. the degradation rate; (b) The time evolution of the concentrations of proteins **LacI **and *λ ****CI ***for *D *= 0.02 and *d *= 1.0.

To obtain the qualitative effect of the external noises on switching, we plot a bifurcation figure [39] [see Fig. 1(a)] by which to anticipate the effect of fluctuations allowed in the degradation rates. For the deterministic system, the numerical results show that for certain (small or large) values of the degradation rate *d *= *d*_1 _= *d*_2 _[also see Fig. 1(a)], the repressors have one unique steady state, whereas for some modest values of *d*, they have three steady states: one is unstable and the other two stable [also see Fig. 1(a)]. To incorporate fluctuations to the deterministic system, we envision *d*_1 _and *d*_2 _to stochastically vary in the bistable region of Fig. 1(a) (see the middle part corresponding to the modest values of *d*). Interestingly, one repressor with high-level expression is more easily fluctuated by the external noise in contrast to the other repressor with low-level expression. Moreover, external noise-induced fluctuations appearing in two repressor concentrations are different: if the fluctuations in one repressor concentration are large, then the fluctuations in the other repressor concentration are small and vice versa [Fig. 1(b)].

Detailed numerical results further verify such an effect of the external noise. For example, if the protein **LacI **is initially in a high state corresponding to the steep top branch in Fig. 1(a), then its concentration is notably fluctuated within the interval [0, 1200] of time. After about 1200 mins, i.e., after the protein drops to the low state, where it has gone through the intermediate state [see the dotted line branch of Fig. 1(a)], the fluctuations in the protein concentration display small deviations from its averaged concentration unless the protein switches to the original high state again. Meanwhile, the concentration of the other repressor protein, *λ ***CI**, alternatively evolves with the reverse fluctuation property in contrast to the protein **LacI **[see Fig. 1(b)]. That is, if the *λ ***CI **concentration is initially at a low level (approximately [*λ ***CI**] = 0.6), then the situation continues until the protein quickly jumps to the high state beginning at about 1200 mins. After that, the *λ ***CI **concentration will fluctuate around this high state. In a word, the fluctuations introduced into the degradation rates are capable of inducing two repressor concentrations to switch from one stable state to the other by crossing their unstable state, but the degree of noise-induced fluctuations in these two repressor concentrations is remarkably different.

**Figure 2:**
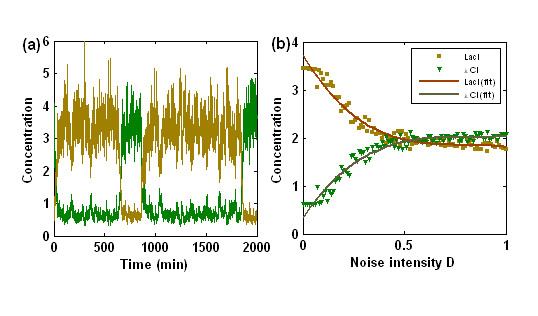
(a) The external noise introduced into the degradation rate can induce successive switching between two stable steady states for a moderate noise intensity *D *= 0.02; (b) The dependence relationship of the protein concentrations, [**LacI**] and [*λ ****CI***], on the external noise intensity *D*, where the solid curves represent fitting results. See the context for parameter settings.

In addition, external noises also have the effect of both inducing a successive switch process between two stable steady states and playing a role of amplifier in the manner of only increasing a protein concentration. To display such an effect clearly, we consider only the case of Gaussian white noises added to the end of Eqs. (3) and (4), with the symmetric repression (only for a clear numerical display): *n*_1 _= *n*_2 _and *α*_1 _= *α*_2_. In this case, we make some modifications for the set-previously parameter values: *n*_1 _= *n*_2 _= 1.6, *α*_1 _= *α*_2 _= 5, *γ*_1 _= *γ*_2 _= 0, and *d*_1 _= *d*_2 _= 1.0. Besides, we change the initial settings for two repressor protein concentrations: [**LacI**] = 3.4 and [*λ ***CI **] = 0.64. In the region of bistability, the time courses of protein **LacI **and *λ ***CI **concentrations are plotted by directly simulating the stochastic differential equation (3) and (4), referring Fig. 2(a). It is shown that as time evolves, **LacI **and *λ ***CI **concentrations alternately fluctuates around their high state and low state, indicating that a successive switch process between two steady states can also be induced by the fluctuation of degradation rates for the modified model parameters. Fig. 2(b) shows the dependence of two repressor protein concentrations on the noise strength *D*, where we take the averaged value of [**LacI**] over a long time for a fixed *D*. Clearly, only the *λ ***CI **concentration increases with the increase but in a finite interval of *D*, whereas the **LacI **concentration instead decreases. On the other hand, if the **LacI **protein is initially in a low state while the *λ ***CI **protein in a high state, then the **LacI **concentration will increase whereas *λ ***CI **concentration instead decreases. Such an amplification effect of the external noises is completely different to that displayed in the case of one-gene autoregulation [21]. This should not be a surprise because two repressor proteins in our case repress each other, leading that only one protein concentration is amplified.

The above numerical results can be simply explained from the viewpoint of physics as follows. For the toggle switch, since the stable fixed points of repressor concentrations correspond to the minima of the "energy landscape" [35], the effect of multiplicative noise is to cause random kicks to the particle (i.e., a system state point) lying in one of these minima. On occasion, a sequence of kicks may enable the particle to escape the local minimum and reside in a new valley. For a nonsymmetric "energy landscape", low noise will enable only transitions from the upper state to the lower state, because random kicks are not sufficient to climb the steep barrier from the lower state [see Fig. 1(b)]. However, for a symmetric "energy landscape", a moderate noise can induce successive transitions between both of the states, as shown in Fig. 2(a). Such a dynamical bistable behavior is impossible in bistable systems based on a classical deterministic description or without the prescribed external noise introduced into degradation terms [40].

### Case 2: Multicellular toggle switch with a quorum-sensing signaling pathway

#### Model

**Figure 3:**
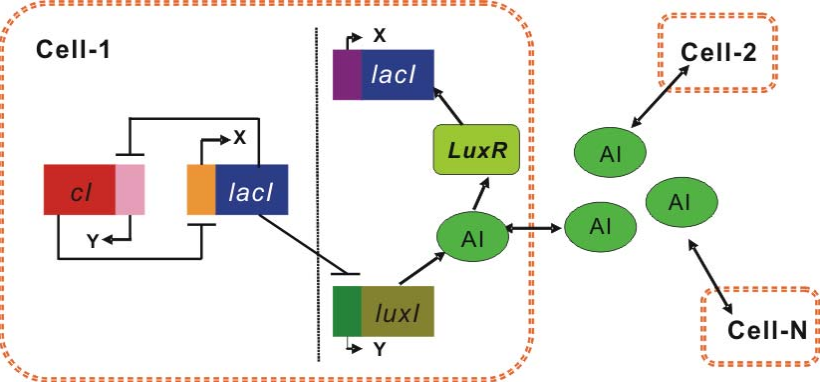
Sketch map for the network system of genetic toggle switches coupled by a quorum-sensing mechanism.

The molecule details of the designed gene regulatory network are illustrated schematically in Fig. 3. This gene network combines two features: the system acts as a toggle switch and uses an intercellular signaling mechanism to couple between cells. The quorum-sensing system from *Vibrio fischeri *[41,42] enables cells to sense population density through a transcription factor protein LuxR which acts as a transcriptional activator of gene *lacI *when a small organic molecule, autoinducer (**AI**), binds to it. The **AI **is synthesized by the protein encoded by the gene *luxI*, which can diffuse across the cell membrane, resulting in two kinds of concentrations: the extracellular concentration of **AI **in the environment, as well as the **AI **concentration in individual cells. They all depend on the density of **AI**-related cells. This property of the quorum-sensing mechanism has been successfully used in the design of some cellular communication systems, where a collective rhythmic behavior across a cell population can be achieved [25,43].

Based on the designed-above gene regulatory network, we develop a stochastic model for a multi-cellular toggle switch system comprising *N *cells in the culture. The dynamics of the proteins **LacI**, *λ ***CI **and **AI **are described as follows:

dxidt=α11+yin1−(d1+ξ1i(t))xi+γ1+βAi1+Ai
 MathType@MTEF@5@5@+=feaafiart1ev1aaatCvAUfKttLearuWrP9MDH5MBPbIqV92AaeXatLxBI9gBaebbnrfifHhDYfgasaacPC6xNi=xI8qiVKYPFjYdHaVhbbf9v8qqaqFr0xc9vqFj0dXdbba91qpepeI8k8fiI+fsY=rqGqVepae9pg0db9vqaiVgFr0xfr=xfr=xc9adbaqaaeGacaGaaiaabeqaaeqabiWaaaGcbaqcfa4aaSaaaeaacqWGKbazcqWG4baEdaWgaaqaaiabdMgaPbqabaaabaGaemizaqMaemiDaqhaaOGaeyypa0tcfa4aaSaaaeaaiiGacqWFXoqydaWgaaqaaiabigdaXaqabaaabaGaeGymaeJaey4kaSIaemyEaK3aa0baaeaacqWGPbqAaeaacqWGUbGBdaWgaaqaaiabigdaXaqabaaaaaaakiabgkHiTiabcIcaOiabdsgaKnaaBaaaleaacqaIXaqmaeqaaOGaey4kaSIae8NVdG3aaSbaaSqaaiabigdaXiabdMgaPbqabaGccqGGOaakcqWG0baDcqGGPaqkcqGGPaqkcqWG4baEdaWgaaWcbaGaemyAaKgabeaakiabgUcaRiab=n7aNnaaBaaaleaacqaIXaqmaeqaaOGaey4kaSscfa4aaSaaaeaacqWFYoGycqWGbbqqdaWgaaqaaiabdMgaPbqabaaabaGaeGymaeJaey4kaSIaemyqae0aaSbaaeaacqWGPbqAaeqaaaaaaaa@5D80@

dyidt=α21+xin2−(d2+ξ2i(t))yi+γ2
 MathType@MTEF@5@5@+=feaafiart1ev1aaatCvAUfKttLearuWrP9MDH5MBPbIqV92AaeXatLxBI9gBaebbnrfifHhDYfgasaacPC6xNi=xI8qiVKYPFjYdHaVhbbf9v8qqaqFr0xc9vqFj0dXdbba91qpepeI8k8fiI+fsY=rqGqVepae9pg0db9vqaiVgFr0xfr=xfr=xc9adbaqaaeGacaGaaiaabeqaaeqabiWaaaGcbaqcfa4aaSaaaeaacqWGKbazcqWG5bqEdaWgaaqaaiabdMgaPbqabaaabaGaemizaqMaemiDaqhaaOGaeyypa0tcfa4aaSaaaeaaiiGacqWFXoqydaWgaaqaaiabikdaYaqabaaabaGaeGymaeJaey4kaSIaemiEaG3aa0baaeaacqWGPbqAaeaacqWGUbGBdaWgaaqaaiabikdaYaqabaaaaaaakiabgkHiTiabcIcaOiabdsgaKnaaBaaaleaacqaIYaGmaeqaaOGaey4kaSIae8NVdG3aaSbaaSqaaiabikdaYiabdMgaPbqabaGccqGGOaakcqWG0baDcqGGPaqkcqGGPaqkcqWG5bqEdaWgaaWcbaGaemyAaKgabeaakiabgUcaRiab=n7aNnaaBaaaleaacqaIYaGmaeqaaaaa@5386@

dAidt=εyi−μAi+σ(Ae−Ai)
 MathType@MTEF@5@5@+=feaafiart1ev1aaatCvAUfKttLearuWrP9MDH5MBPbIqV92AaeXatLxBI9gBaebbnrfifHhDYfgasaacPC6xNi=xI8qiVKYPFjYdHaVhbbf9v8qqaqFr0xc9vqFj0dXdbba91qpepeI8k8fiI+fsY=rqGqVepae9pg0db9vqaiVgFr0xfr=xfr=xc9adbaqaaeGacaGaaiaabeqaaeqabiWaaaGcbaqcfa4aaSaaaeaacqWGKbazcqWGbbqqdaWgaaqaaiabdMgaPbqabaaabaGaemizaqMaemiDaqhaaOGaeyypa0dcciGae8xTduMaemyEaK3aaSbaaSqaaiabdMgaPbqabaGccqGHsislcqWF8oqBcqWGbbqqdaWgaaWcbaGaemyAaKgabeaakiabgUcaRiab=n8aZjabcIcaOiabdgeabnaaBaaaleaacqWGLbqzaeqaaOGaeyOeI0Iaemyqae0aaSbaaSqaaiabdMgaPbqabaGccqGGPaqkaaa@48CC@

dAedt=QN∑i=1N(Ai−Ae)−deAe+Iext(t)
 MathType@MTEF@5@5@+=feaafiart1ev1aaatCvAUfKttLearuWrP9MDH5MBPbIqV92AaeXatLxBI9gBaebbnrfifHhDYfgasaacPC6xNi=xI8qiVKYPFjYdHaVhbbf9v8qqaqFr0xc9vqFj0dXdbba91qpepeI8k8fiI+fsY=rqGqVepae9pg0db9vqaiVgFr0xfr=xfr=xc9adbaqaaeGacaGaaiaabeqaaeqabiWaaaGcbaqcfa4aaSaaaeaacqWGKbazcqWGbbqqdaWgaaqaaiabdwgaLbqabaaabaGaemizaqMaemiDaqhaaOGaeyypa0tcfa4aaSaaaeaacqWGrbquaeaacqWGobGtaaGcdaaeWbqaaiabcIcaOiabdgeabnaaBaaaleaacqWGPbqAaeqaaOGaeyOeI0Iaemyqae0aaSbaaSqaaiabdwgaLbqabaGccqGGPaqkaSqaaiabdMgaPjabg2da9iabigdaXaqaaiabd6eaobqdcqGHris5aOGaeyOeI0Iaemizaq2aaSbaaSqaaiabdwgaLbqabaGccqWGbbqqdaWgaaWcbaGaemyzaugabeaakiabgUcaRiabdMeajnaaBaaaleaacqWGLbqzcqWG4baEcqWG0baDaeqaaOGaeiikaGIaemiDaqNaeiykaKcaaa@55D8@

where *x*_*i *_and *y*_*i *_are the concentrations of **LacI **and *λ ***CI **in cell *i*, respectively; *A*_*i *_is the concentration of **AI **inside the *i*th cell whereas *A*_*e *_is the concentration of **AI **in the extracellular environment. *β *is the maximal contribution to *lacI *transcription of saturating amounts of **AI**, *σ *and *Q *measure the diffusion rate of **AI **across the cell membrane, *ε *and *μ *are the synthesis and degradation rates of the intracellular **AI**, respectively, and *d*_*e *_is the extracellular **AI **degradation rate. *ξ*_*ki*_(*t*) represent intracellular noises due to stochastic fluctuations in the process of degradation inside cells, and are assumed as independently and identically distributed Gaussian white noises with <*ξ*_*ki*_(*t*)> = 0 and <*ξ*_*ki*_(*t*)*ξ*_*ki*_(*t'*)> = *Dδ*_*i*, *j*_(*t*-*t*'), *k *= 1,2. These *ξ*_*ki*_(*t*) constitute multiplicative intracellular noises to every toggle switch. *I*_*ext*_(*t*) represents an extracellular stimulus [44]. We will consider two cases: (1) *I*_*ext*_(*t*) = *A*sin(Ω*t*); (2) *I*_*ext*_(*t*) = *A*sin(Ω*t*) + *ζ*(*t*). In both cases, the term *A*sin(Ω*t*) can be viewed as a periodical forcing or stimulus injected in the extracellular environment [22,45]. Analogy of introducing such a term is in the study of behaviors of circadian rhythms (i.e., biochemical rhythms with a period close to 24 hours that have been observed ubiquitously among living organisms) [46]. In many organisms, the source of external forcing has been identified to be a variation of the light due to night and day cycles. Recently, the molecular basis about the effect of the light on different circadian biochemical networks has been unraveled [47]. In addition, the term *A*sin(Ω*t*) was also ever introduced into a genetic relaxation oscillator (see explanations in Ref. [22]). In the second case, the term *ζ*(*t*) is called as extracellular noise, since it originates outside the cells due to environment perturbations, and is also assumed as the Gaussian white noise with the autocorrelation function <*ζ*(*t*)*ζ*(*t'*)> = *D*_*ext*_*δ*(*t*-*t*'), where *D*_*ext *_stands for noise intensity. In addition, it is reasonable to assume that *ζ*(*t*) is uncorrelated with *ξ*_*i*_(t) since the intercellular noises in a cell are generally irrelative to the extracellular noises and vice versa. On model structure, here we point out that systems (5)-(8) is different from that multicellular model for the toggle switch system developed in [19,29], although they all are an interacting system through a quorum-sensing pathway. The main difference is in the description of the expression of the gene that encodes **LuxI**. In [29], the synthesis rate of **AHL **is a combination of the expression rate of the houskeeping gene *luxI *and the synthesis rate of **AHL **from **LuxI **and is set to a constant *b*_1_. Here, in the spirit of ref. [42], we propose to incorporate this quorum-sensing signaling apparatus into the genetic toggle switch by placing the gene that encodes **LuxI **under the control of the protein **LacI **[25,48], as shown in Fig. 3 and described by Eq. (7).

In the following, we fix parameters: *β *= 15, *σ *= 10, *ε *= 0.07, *μ *= 1, *Q *= 0.5, *d*_*e *_= 3, *A *= 0.08 and Ω = 2*π*/400. We will investigate the effect of noises, in particular the effect of interplay between extracellular stimuli and noises described by *D *or *D*_*ext*_, on synchronized switching.

#### Results

##### 1. Intracellular noises can induce synchronized switching

Although two stochastic models (where only internal noises are considered) that have been used to realize experimental results with bimodal population distributions [6,19] for the toggle switch system interfaced with either the SOS or the quorum-sensing signaling pathway have been proposed [29], how external noise source introduced into the degradation rate affects collective switching in a multicellular toggle switch network coupled by quorum sensing still remains unclear. In this subsection, we will show that the intracellular noise can induce a stochastic resonance, i.e., synchronized switching, across a population of the cells. In particular, when the noise is interacted with an injected sinusoidal signal (or stimulus) in the extracellular environment, we expect that such an effect of the noise becomes more notable, leading a robust collective rhythm.

To quantify how good the synchronized switching is, we calculate an order parameter [48,49] by

**Figure 4:**
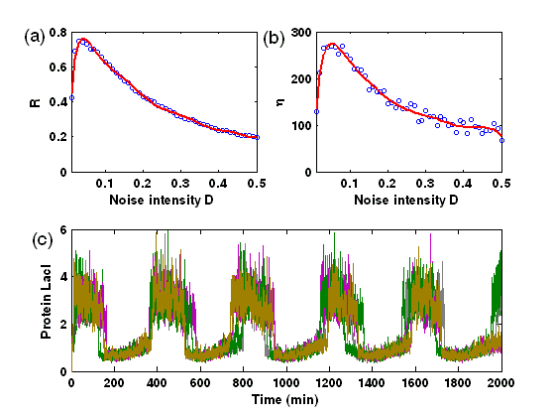
The effect of intracellular noise intensity *D *on the synchronized switching in multicellular toggle switch systems coupled by quorum sensing. (a) Quantified by the order parameter R; (b) Shown by the amplification factor *η*; (c) Displayed by the time evolution of **LacI **concentrations of five cells for 100 cells at *D *= 0.05, where intracellular noise-induced synchronized switching is clearly observed.

R=<M2>−<M>2<xi2>−<xi>2¯
 MathType@MTEF@5@5@+=feaafiart1ev1aaatCvAUfKttLearuWrP9MDH5MBPbIqV92AaeXatLxBI9gBaebbnrfifHhDYfgasaacPC6xNi=xI8qiVKYPFjYdHaVhbbf9v8qqaqFr0xc9vqFj0dXdbba91qpepeI8k8fiI+fsY=rqGqVepae9pg0db9vqaiVgFr0xfr=xfr=xc9adbaqaaeGacaGaaiaabeqaaeqabiWaaaGcbaGaemOuaiLaeyypa0tcfa4aaSaaaeaacqGH8aapcqWGnbqtdaahaaqabeaacqaIYaGmaaGaeyOpa4JaeyOeI0IaeyipaWJaemyta0KaeyOpa4ZaaWbaaeqabaGaeGOmaidaaaqaamaanaaabaGaeyipaWJaemiEaG3aa0baaeaacqWGPbqAaeaacqaIYaGmaaGaeyOpa4JaeyOeI0IaeyipaWJaemiEaG3aaSbaaeaacqWGPbqAaeqaaiabg6da+maaCaaabeqaaiabikdaYaaaaaaaaaaa@456C@

where M(t)=1N∑i=1Nxi(t)
 MathType@MTEF@5@5@+=feaafiart1ev1aaatCvAUfKttLearuWrP9MDH5MBPbIqV92AaeXatLxBI9gBaebbnrfifHhDYfgasaacPC6xNi=xH8viVGI8Gi=hEeeu0xXdbba9frFj0xb9qqpG0dXdb9aspeI8k8fiI+fsY=rqGqVepae9pg0db9vqaiVgFr0xfr=xfr=xc9adbaqaaeGacaGaaiaabeqaaeqabiWaaaGcbaGaemyta0KaeiikaGIaemiDaqNaeiykaKIaeyypa0tcfa4aaSaaaeaacqaIXaqmaeaacqWGobGtaaGcdaaeWbqaaiabdIha4naaBaaaleaacqWGPbqAaeqaaOGaeiikaGIaemiDaqNaeiykaKcaleaacqWGPbqAcqGH9aqpcqaIXaqmaeaacqWGobGta0GaeyyeIuoaaaa@40C1@ (*N*: the number of cells), < · > denotes the averaging over time, and <⋅⋅⋅>¯
 MathType@MTEF@5@5@+=feaafiart1ev1aaatCvAUfKttLearuWrP9MDH5MBPbIqV92AaeXatLxBI9gBaebbnrfifHhDYfgasaacPC6xNi=xH8viVGI8Gi=hEeeu0xXdbba9frFj0xb9qqpG0dXdb9aspeI8k8fiI+fsY=rqGqVepae9pg0db9vqaiVgFr0xfr=xfr=xc9adbaqaaeGacaGaaiaabeqaaeqabiWaaaGcbaWaa0aaaeaacqGH8aapcqGHflY1cqGHflY1cqGHflY1cqGH+aGpaaaaaa@34CE@ indicates averaging over all cells. In this way, *R *≈ 1 in the synchronized regime whereas *R *≈ 0 in the unsynchronized regime. Note that such an order parameter *R *is originally used to describe the synchronization degree in coupled limit-cycle oscillators [48], but it is still effective in describing the synchronized switching induced by the intracellular noises in the case of bistable systems. Numerical results indicate that in our case, the effect of the synchronized switching has been quite good when *R *is larger than 0.6. Fig. 4(a) plots the dependence relationship of the order parameter *R *on the noise strength *D *for an ensemble of *N *(= 100) cells. For this figure, we emphasize two points: (1) There is an large interval of *D*, such that the values of the corresponding *R *are beyond 0.6, implying that the synchronized switching has been attained. For example, *R *> 0.6 when 0.015 ≤ *D *≤ 0.12 [Fig. 4(a)]; (2) *R *has the maximal value at a certain value of *D*, e.g., *R *≈ 0.78 at *D *≈ 0.05 [Fig. 4(a)]. In other words, there is an optimal noise level such that the order parameter *R *reaches its maximum, similar to that in the usual noise-induced coherent resonance. In addition, for such a *D *with the maximal *R*, the ensemble of cells show phase synchronization with phase slip [see Fig. 4(c)]. The above result indicates a novel constructive role of intracellular noise in an indirectly coupled toggle switch system.

To measure the relative performance of switching quantitatively, we also calculate the spectral amplification factor *η*, which is, according to Ref. [50], defined as

*η *= 4*A*^-2 ^|<*e*^*i*Ω*t *^*M*(*t*)>|^2 ^

where M(t)=1N∑i=1Nxi(t)
 MathType@MTEF@5@5@+=feaafiart1ev1aaatCvAUfKttLearuWrP9MDH5MBPbIqV92AaeXatLxBI9gBaebbnrfifHhDYfgasaacPC6xNi=xH8viVGI8Gi=hEeeu0xXdbba9frFj0xb9qqpG0dXdb9aspeI8k8fiI+fsY=rqGqVepae9pg0db9vqaiVgFr0xfr=xfr=xc9adbaqaaeGacaGaaiaabeqaaeqabiWaaaGcbaGaemyta0KaeiikaGIaemiDaqNaeiykaKIaeyypa0tcfa4aaSaaaeaacqaIXaqmaeaacqWGobGtaaGcdaaeWbqaaiabdIha4naaBaaaleaacqWGPbqAaeqaaOGaeiikaGIaemiDaqNaeiykaKcaleaacqWGPbqAcqGH9aqpcqaIXaqmaeaacqWGobGta0GaeyyeIuoaaaa@40C1@, and < · > denotes averaging over time. In Fig. 4(b), we plot the amplification factor *η *versus the noise intensity *D *for a given amplitude *A *of the external forcing. By comparing Fig. 4(b) with Fig. 4(a), we find that the amplification factor curve for *η *vs. *D *displays the characteristic maximum at a critical intracellular noise value that is nearly the same as the value of the noise at which the curve of order parameter *R *vs. *D *also displays the characteristic maximum taken as the hallmark of synchronized switching.

We further point out that *R *in Fig. 4(a) is mainly used to describe the synchronization degree among the toggle switch systems while *η *in Fig. 4(b) to show the effect of coherent switching, and they altogether are used to describe synchronized switching. More preciously, for the former, the synchronization effect is optimal at the maximal *R *(≈ 0.78, approximately at *D *= 0.05), referring Fig. 4(c); For the latter, it means that the larger the *η*, the better the switching, e.g., when *D *is approximately equal to 0.05, *η *approaches its maximum [see Fig. 4(b)], and in this case, the switching becomes notable [see Fig. 4(c)], but the time evolution of the protein **LacI **concentrations is not shown here for other values of *D*.

##### 2. Extracellular noise can induce synchronized switching

Different from the previous subsection in which the effect of intracellular noise on switching has been investigated, this subsection will mainly focus on the effect of common extracellular noise on synchronized switching. Assume that

*I*_*ext *_= *A*sin(Ω*t*) + *ζ*(*t*)

For simplicity, we further assume that there is no intracellular noise. In other words, only the extracellular noise is considered in this subsection. In this case, to quantify the level of synchronized switching across a population of cells, we introduce the averaged synchronization error (*ASE*) which is, according to [51], defined as

**Figure 5:**
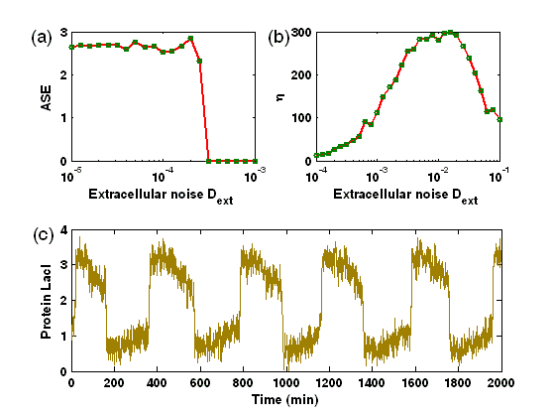
The effect of extracellular noise intensity *D*_*ext *_on the synchronized switching in multicellular toggle switch systems coupled by quorum sensing. (a) Quantified by the average synchronization error (ASE); (b) Shown by the amplification factor *η*; (c) Displayed by the time evolution of **LacI **concentrations of five cells for 100 cells at *D *= 0.005, where extracellular noise-induced synchronized switching is clearly observed.

ASE=〈1CN2∑i>j[xi−xj]2〉
 MathType@MTEF@5@5@+=feaafiart1ev1aaatCvAUfKttLearuWrP9MDH5MBPbIqV92AaeXatLxBI9gBaebbnrfifHhDYfgasaacPC6xNi=xI8qiVKYPFjYdHaVhbbf9v8qqaqFr0xc9vqFj0dXdbba91qpepeI8k8fiI+fsY=rqGqVepae9pg0db9vqaiVgFr0xfr=xfr=xc9adbaqaaeGacaGaaiaabeqaaeqabiWaaaGcbaGaemyqaeKaem4uamLaemyrauKaeyypa0ZaaaWaaeaajuaGdaWcaaqaaiabigdaXaqaaiabdoeadnaaDaaabaGaemOta4eabaGaeGOmaidaaaaakmaaqafabaGaei4waSLaemiEaG3aaSbaaSqaaiabdMgaPbqabaGccqGHsislcqWG4baEdaWgaaWcbaGaemOAaOgabeaakiabc2faDnaaCaaaleqabaGaeGOmaidaaaqaaiabdMgaPjabg6da+iabdQgaQbqab0GaeyyeIuoaaOGaayzkJiaawQYiaaaa@47A8@

where CN2
 MathType@MTEF@5@5@+=feaafiart1ev1aaatCvAUfKttLearuWrP9MDH5MBPbIqV92AaeXatLxBI9gBaebbnrfifHhDYfgasaacPC6xNi=xH8viVGI8Gi=hEeeu0xXdbba9frFj0xb9qqpG0dXdb9aspeI8k8fiI+fsY=rqGqVepae9pg0db9vqaiVgFr0xfr=xfr=xc9adbaqaaeGacaGaaiaabeqaaeqabiWaaaGcbaGaem4qam0aa0baaSqaaiabd6eaobqaaiabikdaYaaaaaa@2F26@ = *N*(*N*-1)/2 represents the combinational number of arbitrarily chosen two components among *N *components, the sum of squared differences is taken over a large time interval after some initial transients are discarded, and < · > denotes averaging over time. When all the cells have the completely synchronized dynamics, the average of the errors over the cells should be zero, i.e., *ASE *≈ 0. Otherwise, *ASE *will be more than zero. The plot for the synchronization error *ASE *vs. the extracellular noise strength *D*_*ext *_is shown in Fig. 5(a) for an ensemble of *N *(= 100) cells. Clearly, there exists a sudden change between two limiting values of *ASE*, implying that a transition to synchronized switching has occurred. Moreover, the synchronized switching exists for an interval of *D*_*ext *_beginning at *D*_*ext *_≈ 0.00015. In addition, since the amplification factor *η *as defined above can be used to describe the degree of switching, we further calculate the *η*. Fig. 5(b) shows the dependence relationship of the amplification factor *η *on the extracellular noise intensity *D*_*ext*_, where there is an optimal *D*_*ext *_at which *η *has a maximal value, implying that a coherent resonance has taken place. The temporal courses of the protein **LacI **concentration corresponding to the case of *D*_*ext *_≈ 0.005 are plotted in Fig. 4(c), displaying a notable synchronized switching.

Note that we here adopt the averaged synchronization error *ASE *instead of the order parameter *R *to describe the degree of synchronization. In fact, our numerical simulations have verified that the order parameter would be invalid in describing synchronized switching in the presence of only extracellular noise and no intracellular noise, e.g., it is possible that *R *approaches to 1 in this case, but the synchronized switching are not achieved (data are not shown here). This would be because the effect of the extracellular noise is averaged to distribute into every cell through the signaling molecule, leading that the corresponding components of arbitrary two cells has the closer correlation which thus produces a supposititious order parameter for describing the synchronization effect. However, in the case of intracellular noises, the situation is different. Since the intracellular noises have been in advance assumed to be independent each another, such an independence would lead a tendency which makes the corresponding components between distinct cells deviate stochastically. Nevertheless, a remarkable synchronized switching induced by common extracellular noise can occur in an ensemble of the toggle switch systems interfaced by a quorum-sensing signaling pathway [Fig. 5(c)].

Generally, extracellular noise is common to all cells because it results from the perturbation of the cellular environment. Consequently, it may have the active effect of synchronizing the dynamics of all the cells by exerting the identical fluctuation on each cell through signaling molecules. The results presented in this subsection have demonstrated that the extracellular noise indeed can induce the synchronized switching, generating a significant resonance phenomenon where the maximum of the amplification factor corresponds to a preferred stochastic oscillation. Although it is not easy to directly control the extracellular noise intensity for achieving the most pronounced synchronized switching, we expect that biological systems possibly use some regulatory mechanisms to optimally facilitate their functions during internal cellular processes.

##### 3. Extracellular noise can enhance synchronized switching

In this subsection, we will investigate the effect of the extracellular noise on the synchronized switching in the presence of the intracellular noises, especially interested in the case that the synchronized switching cannot be achieved only by the intracellular noise. Note, in this case, that there are two kinds of noises in model: intracellular noise *ξ*_*i*_(*t*) (*i *= 1,2) and extracellular noise *ζ*(*t*). There have been few results on the effect of noises in the simultaneous presence of two kinds of noises. Zhou [25] and Chen [52] investigated the effect of extracellular noise on cellular communication for a given intracellular noise. They showed that without extracellular noises, all cells, in spite of interaction among them, behave irregularly due to independent intracellular noises, and on the other hand, extracellular noises that are common to all cells can induce collective dynamics and stochastically synchronize the multi-cell system by actively enhancing the integrated interchange of signaling molecules. Now, we numerically analyze the effects of these two kinds of noises on synchronized switching behaviors in systems (5)-(8).

**Figure 6:**
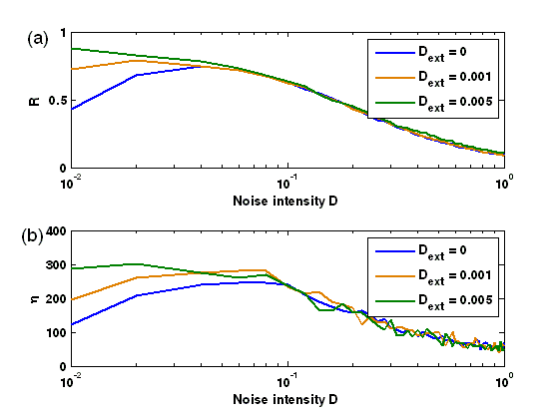
The extracellular noise can enhance the synchronized switching in the multicellular toggle switch system, by comparing two quantities: (a) The order parameter R; and (b) The amplification factor *η*.

**Figure 7:**
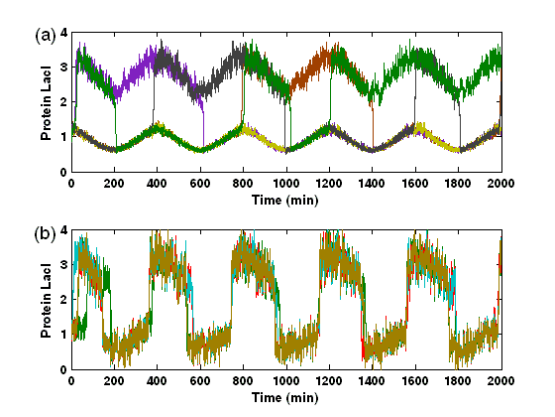
An example showing that extracellular noise can induce/enhance the synchronized switching in the case that intracellular noise cannot induce a synchronized switching, where *D *= 0.005. (a) The time evolution of concentrations of five **LacI **proteins for 100 cells at *D*_*ext *_= 0, where the synchronized switching is not achieved; (b) The time evolution of concentrations of five **LacI **proteins for 100 cells at *D*_*ext *_= 0.006, where the synchronized switching is achieved.

Figure 6 shows the relationship between the order parameter *R *and the amplification factor *η *and the intracellular noise strength *D*, respectively, for several different strengths of extracellular noise. Clearly, for some fixed low-level intracellular noises (e.g., *D *= 0.005), *R *becomes larger in the presence of extracellular noise [see the curves corresponding to *D*_*ext *_= 0.001 and 0.005 in Fig. 6(a), respectively] than in the case of no extracellular noise, i.e., *D*_*ext *_= 0, indicating that the extracellular noise can enhance synchronized switching. Moreover, *R *increases but is within a small interval as the extracellular noise strength increases. The similar results also hold for the amplification factor *η*, indicating that the extracellular noise has another effect, namely it can amplify the extracellular signaling. Such an effect of the extracellular noise, i.e., enhancing stochastic synchronized switching, is further verified, as shown in Fig. 7. Without extracellular noise, an ensemble of cells cannot achieve synchronized switching at some intracellular noise strength [Fig. 7(a)]. On the other hand, an appropriate extracellular noise can induce synchronized switching [Fig. 7(b)]. By comparing Fig. 7(c) and Fig. 7(b), we find that the effect of synchronized switching induced by the extracellular noise plus some given intracellular noises is better than that of synchronized switching induced only by intracellular noise. This would be because extracellular noise has the effect of suppressing intracellular noises.

Extracellular noise in this context means stochastic variation in the environmental distribution of the autoinducer. The first model for cell-cell communication system was proposed by Zhou [25], which possesses both intracellular and extracellular sources of noise. They showed that the noise from extracellular medium can act as a compensating signal source to enhance an integrated exchange of information and force all the cells to be stochastically synchronized. As pointed by Springer and Paulsson [53], the result in Ref. [25] profoundly changed the perception of the role of noise in quorum sensing and in biological systems in general. Here, based on a multicellular toggle switch network coupled by quorum sensing, we reproduced the constructive role of extracellular noise in enhancing the synchronized switching.

## Discussion

Bistability and switching are two important aspects of many genetic regulatory networks. It has been shown that in the genetic toggle switch system, transitions from one stable steady state to the other can be induced by a signal that temporarily brings the system out of the region of bistability [19,29]. On the other hand, gene regulatory network is an inherent noisy process, from transcriptional control, alternative splicing, translation, diffusion to chemical modification reactions of transcriptional factors, which all involve stochastic fluctuations due to low copy numbers of species per cell. Based on network systems of a bistable toggle switch and by introducing stochastic kinetic models for describing the effect of external noises from environmental fluctuations, we have shown that these noises all can induce switching from one stable state to the other. In this manner, they play a constructive role for the coordinated behaviors of cells.

The effects of noise and heterogeneity are of particular interest. Previous experimental works have shown that the genetic toggle switch comprising two genes and regulated by a double-negative feedback loop is a robust bistable system in which noise can seldom induce transitions between two stable steady states [6]. However, because of complexity of noise source that would lead to introducing different stochastic models for analysis, the effect of noise is far from being so simple. Gene expression levels in cells exhibit fluctuations, often attributed to the relatively small particle numbers involved [9]. It is possible to argument the rate equations with stochastic terms capturing these fluctuations [54]. In this paper, by introducing stochastic fluctuations for degradation rates, we have shown that the external noises resulting from these fluctuations all can induce switching from one stable steady state to the other.

Tian and Burrage [29] have introduced two stochastic models by applying the Poisson *τ*-leap method: The one is the toggle switch with the SOS pathway; the other is the toggle switch with a quorum-sensing signaling pathway. In particular, for the latter model, they have showed that a successful switching can be achieved for a certain number of the cells and for some Hill functions. This indicates that noise is capable of inducing the switching in a population of cells. In the paper, we have investigated the effect of different types of noises, e.g., additive or multiplicative noises, in an ensemble of toggle switch systems coupled by a quorum-sensing mechanism, and shown that there is an optimal noise strength such that a coherent switching is optimally achieved, especially notable in the case of an extracellular signaling injected into the common cellular environment. Hasty *et al*. [21] have also investigated the role of multiplicative noises on the switching in the autoregulatory network system of one gene. They have shown how these multiplicative external noises can be used to regulation expression and that small deviations in the transcription rate can lead to large fluctuations in the production of protein. In addition, they have also described how these fluctuations can used to amplify protein production significantly. In contrast to that model they considered, our model is different in the way of introducing noises, but we have shown that noises under our considerations have biological roles similar to those obtained by Hasty, *et al*. [21], i.e., they can not only induce switching even synchronized switching but also play an amplifier for gene expression. In spite of the difference in model, it would be interesting to analytically derive some conditions on the relationship between the noise strength (*D*) and the diffusion rate of the signaling molecule across the cell membrane [i.e., *σ *in Eqs. (7)], under which the coherent switching can be optimally achieved. For the toggle switch interfaced by a quorum-sensing signaling pathway, intracellular and extracellular noises have the similar effect but also have the different effect on synchronized switching. First, both all can induce the synchronized switching; second, the independent intracellular noises, on the one hand, have the tendency that the corresponding components of arbitrary two cells would be stochastically deviated, and on the other hand, also can force every toggle switch cell to switch between two states in aid of common extracellular signaling. In contrast to the effect of intracellular noises, that the extracellular noise induces the synchronized switching is by equally exerting its effect into every cell which makes the corresponding components of arbitrary two cells have a closer correlation; Third, in the presence of intracellular and extracellular noises, the extracellular noise can induce/enhance the synchronized switching only when its strength is beyond a threshold. More preciously, for a fixed intracellular noise strength, the extracellular noise exerts its effect on system switching only when its strength is strong enough, and otherwise, the dormant role is the original intracellular noise. In the case that the intracellular noise has induced the synchronized switching, some extracellular noise only enhances such an ordering behavior. It would be, however, interesting to quantitatively find such a threshold of the extracellular noise strength for which the extracellular noise induces and/or enhance the synchronized switching for a fixed intracellular noise.

Finally, the control of cellular functions through the design and manipulation of gene regulatory networks is an intriguing perspective in applications. The results in this paper suggest that the external noise source may be used as a switch for gene expression or intercellular communication. Since current gene therapy techniques are limited in that transfected genes are typically either in an "on" or "off" state [21], the expression of a transfected gene needs to be regulated in a systematic fashion for the effective treatment of many diseases. Thus, the development of noise-based switches for gene expression could have significant clinical implications.

## Conclusion

Based on the famous genetic toggle switch system, the role of external noises in inducing switching and synchronized switching is systemically studied in this paper. We mainly concentrated on two models, i.e., the single and coupled genetic toggle switch systems in *Escherichia coli*. For both cases, our numerical results showed that the multiplicative noises resulting from stochastic fluctuations in degradation rates can induce switching. More importantly, for the network of toggle switches interfaced by a quorum-sensing signaling pathway, we found that intracellular noises, i.e., stochastic fluctuations in degradation rates inside cells, can induce synchronized switching, whereas the extracellular noise additive to the common medium can not only entrain all the individual systems to switch in a synchronous manner but also enhance this ordering behavior efficiently, leading a robust collective rhythm in this interacting system. These insights on the effect of noises would be beneficial to understanding the basic mechanism of how living systems optimally facilitate to function under various fluctuated environments. We hope the phenomena reported here can be further investigated experimentally, to deepen our understanding on the role of noise in switching behaviors.

## Authors' contributions

TZ conceived and designed experiments. JZ, ZY and JW performed the numerical experiments and analyzed the data. JW and TZ wrote the paper. All authors have read and approved the final manuscript.
